# *Cacna1c* haploinsufficiency leads to pro-social 50-kHz ultrasonic communication deficits in rats

**DOI:** 10.1242/dmm.034116

**Published:** 2018-06-20

**Authors:** Theresa M. Kisko, Moria D. Braun, Susanne Michels, Stephanie H. Witt, Marcella Rietschel, Carsten Culmsee, Rainer K. W. Schwarting, Markus Wöhr

**Affiliations:** 1 Behavioral Neuroscience, Experimental and Biological Psychology, Faculty of Psychology, Philipps-University of Marburg, Gutenbergstr. 18, D-35032 Marburg, Germany; 2 Institute of Pharmacology and Clinical Pharmacy, Philipps-University of Marburg, Karl-von-Frisch-Str. 1, D-35032 Marburg, Germany; 3 Department of Genetic Epidemiology in Psychiatry, Central Institute of Mental Health, Faculty of Medicine Mannheim, University of Heidelberg, J5, D-65189 Mannheim, Germany; 4 Center for Mind, Brain, and Behavior (CMBB), Philipps-University of Marburg, Hans-Meerwein-Str. 6, D-35032 Marburg, Germany

**Keywords:** Ca_v_1.2, Calcium, Autism, Social behavior, Rough-and-tumble play, Ultrasonic vocalizations

## Abstract

The cross-disorder risk gene *CACNA1C* is strongly implicated in multiple neuropsychiatric disorders, including autism spectrum disorder (ASD), bipolar disorder (BPD) and schizophrenia (SCZ), with deficits in social functioning being common for all major neuropsychiatric disorders. In the present study, we explored the role of *Cacna1c* in regulating disorder-relevant behavioral phenotypes, focusing on socio-affective communication after weaning during the critical developmental period of adolescence in rats. To this aim, we used a newly developed genetic *Cacna1c* rat model and applied a truly reciprocal approach for studying communication through ultrasonic vocalizations, including both sender and receiver. Our results show that a deletion of *Cacna1c* leads to deficits in social behavior and pro-social 50-kHz ultrasonic communication in rats. Reduced levels of 50-kHz ultrasonic vocalizations emitted during rough-and-tumble play may suggest that *Cacna1c* haploinsufficient rats derive less reward from playful social interactions. Besides the emission of fewer 50-kHz ultrasonic vocalizations in the sender, *Cacna1c* deletion reduced social approach behavior elicited by playback of 50-kHz ultrasonic vocalizations. This indicates that *Cacna1c* haploinsufficiency has detrimental effects on 50-kHz ultrasonic communication in both sender and receiver. Together, these data suggest that *Cacna1c* plays a prominent role in regulating socio-affective communication in rats with relevance for ASD, BPD and SCZ.

This article has an associated First Person interview with the first author of the paper.

## Abstract (precis)

**Summary:** The present study suggests that *Cacna1c* plays a prominent role in regulating socio-affective communication in rats with relevance for neuropsychiatric disorders.

## INTRODUCTION

The cross-disorder risk gene *CACNA1C* is strongly implicated in multiple neuropsychiatric disorders, including autism spectrum disorder (ASD), bipolar disorder (BPD) and schizophrenia (SCZ) (Ferreira et al., 2008; Green et al., 2010; Nyegaard et al., 2010; Splawski et al., 2004). *CACNA1C* codes for the α1C subunit of the voltage-gated L-type calcium channel (LTCC) Ca_v_1.2, regulating depolarization-dependent calcium influx into the cell. Ca_v_1.2 accounts for the majority of all LTCCs in the brain. It plays a pivotal role in regulating neuronal excitability, synaptic plasticity and gene expression, and thus represents a primary therapeutic target (Zamponi, 2016).

Deficits in social functioning, such as failure of normal back-and-forth conversation and abnormal social approach, are common for all major neuropsychiatric disorders (Meyer-Lindenberg and Tost, 2012) and genetic *Cacna1c* mouse models display prominent alterations in social behavior (Kabir et al., 2016). While mice currently tend to be the most commonly used model species, rats have several advantages (Ellenbroek and Youn, 2016). Benefits include genetic variability and overall behavioral richness, which may improve translational validity, particularly when it comes to studies on social behavior and communication (Homberg et al., 2017). Rats are highly gregarious animals with a rich and complex social behavior repertoire. For instance, they display cooperation, reciprocity and mutual reward preferences (Hernandez-Lallement et al., 2015), linked to empathy-driven helping behavior (Ben-Ami Bartal et al., 2011). Importantly, rats begin interacting socially at a very young age and juveniles engage in high levels of rough-and-tumble play behavior, making it the most used model species to study social play (Vanderschuren et al., 2016). The complex nature of social play involves coordination and integration of behavior and communication, requiring numerous neural systems (Vanderschuren et al., 2016), and individual rough-and-tumble play components, such as pinning, wrestling and chasing, were found to be selectively affected by genetic (Homberg et al., 2007), prenatal (Raza et al., 2015), pharmacological (Vanderschuren et al., 1995) and brain (Schneider and Koch, 2005) manipulations.

Acoustic communication is another important component of their social behavior repertoire. Rats emit whistle-like calls in the ultrasonic range, i.e. ultrasonic vocalizations (USVs) (Brudzynski, 2013). Evidence from selective breeding, devocalization and playback studies suggests that the various USV types serve as situation-dependent socio-affective signals fulfilling distinct communicative functions. Specifically, 50-kHz USVs are thought to reflect a positive affective state (‘rat laughter’) (Panksepp, 2005) as they occur in appetitive situations, most notably during and in anticipation of rough-and-tumble play (Knutson et al., 1998), and are required to maintain playful mood (Kisko et al., 2015). They serve important pro-social communicative functions and 50-kHz USV playback induces social approach behavior in receivers by eliciting the anticipation of rewarding social interactions, suggesting that approach evoked by 50-kHz USVs can be used as a behavioral readout for the incentive salience of social contact (Engelhardt et al., 2017).

## RESULTS

**Fig. 1.**
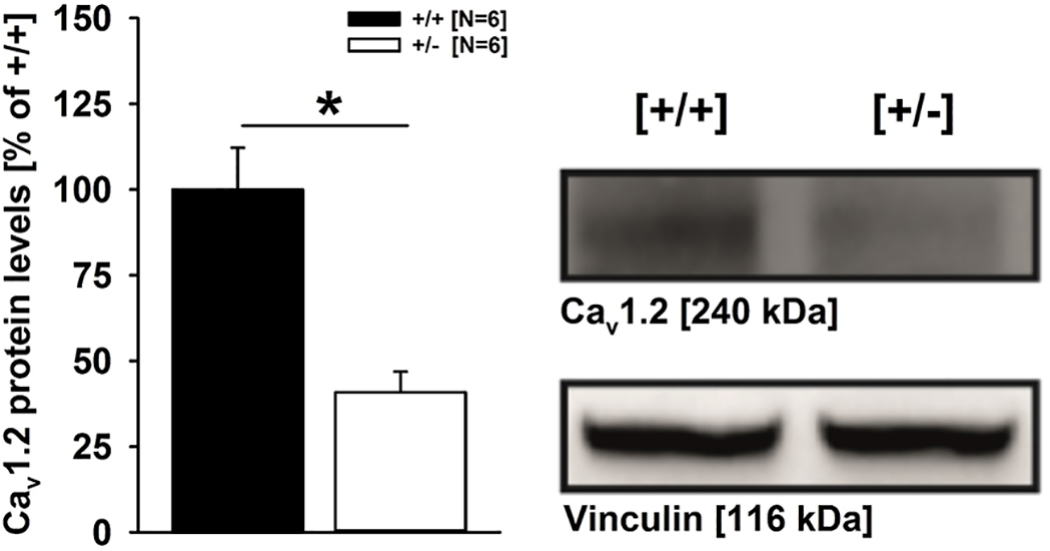
**Ca_V_1.2 protein levels in *Cacna1c^+/^******^−^*** **rats and *Cacna1c^+/+^* littermate controls.** Ca_V_1.2 expression levels were analyzed by western blot from cortical tissue of male *Cacna1c*^+/^*^−^* rats (white bar; *N*=6) and *Cacna1c^+/+^* littermate controls (black bar; *N*=6). The bar graph (left panel) was obtained by densitometric quantification of the western blot data. The results are expressed as percentage of *Cacna1c^+/+^* littermate control values after normalization to the loading control vinculin. The Ca_V_1.2 level of *Cacna1c^+/+^* littermate controls is set as 100%. The immunoblots (right panel) show one representative example per genotype. Data are presented as mean±s.e.m. *P<0.050 vs *Cacna1c^+/+^* littermate controls.

In the present study, we explored the role of *Cacna1c* in regulating behavioral phenotypes, focusing on socio-affective communication after weaning during the critical developmental period of adolescence in rats. To this aim, we used a newly developed genetic *Cacna1c* rat model and applied a truly reciprocal approach for studying communication through pro-social 50-kHz USVs, including both sender and receiver. Effects of *Cacna1c* haploinsufficiency were assessed in male constitutive heterozygous *Cacna1c^+/−^* rats (*N*=20) and compared to wild-type *Cacna1c^+/+^* littermate controls (*N*=20). *Cacna1c^+/−^* rats were generated using zinc-finger technology (for details, see Materials and Methods). As shown by western blot using cortical tissue, Ca_v_1.2 protein levels in *Cacna1c^+/−^* rats are reduced by slightly more than 50% in the brain, as compared to *Cacna1c^+/+^* littermates (*t*_10_=4.345, *P*=0.001; Fig. 1; for representative western blot and antibody validation, see Fig. S1).

### Rough-and-tumble play and pro-social 50-kHz USVs

**Fig. 2.**
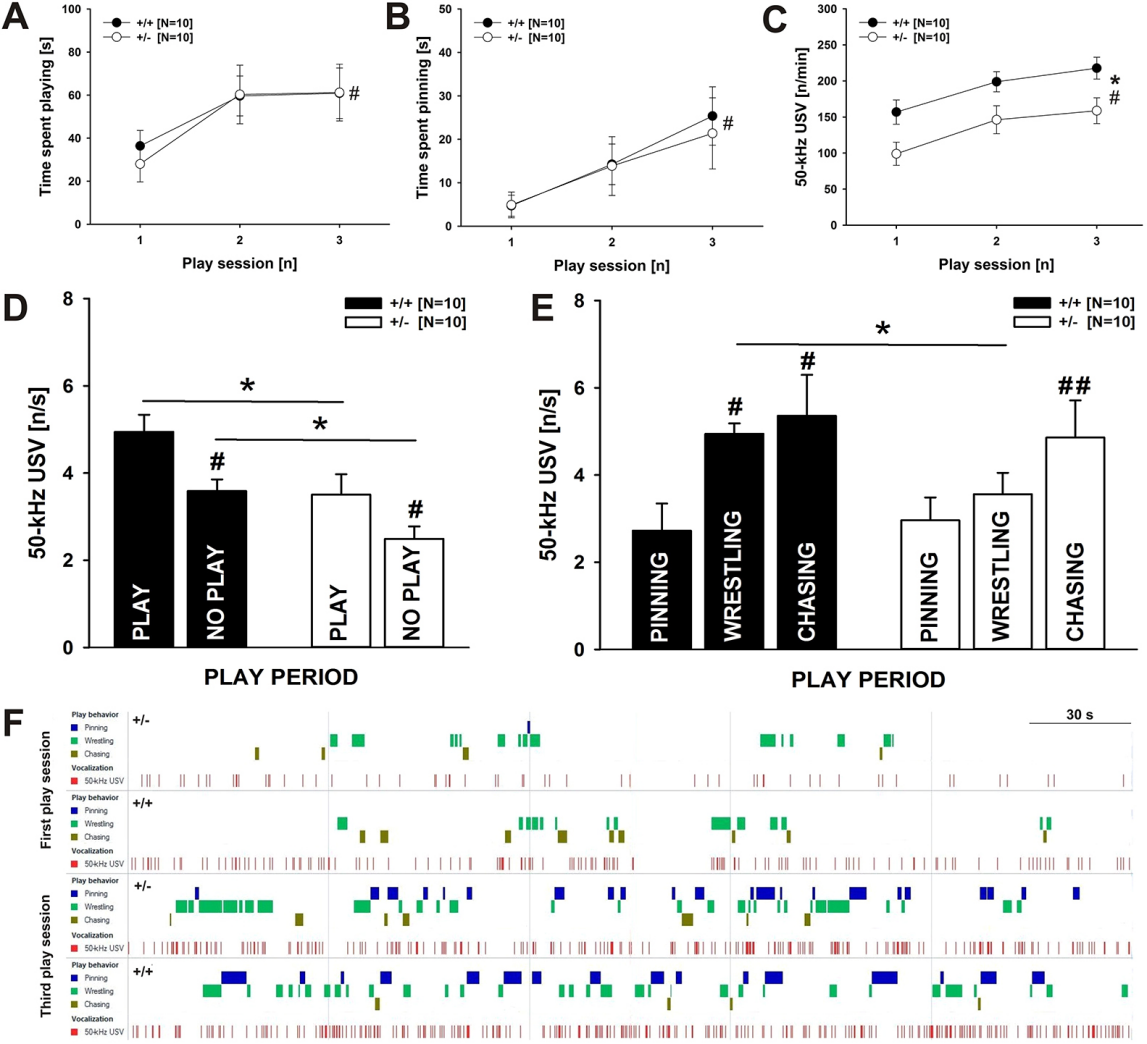
**Rough-and-tumble play behavior and concomitant pro-social 50-kHz USV emission in *Cacna1c*^+/^***^**−**^* **rats and *Cacna1c^+/+^* littermate controls.** (A) Time spent playing, (B) time spent pinning and (C) 50-kHz USV emission across the three play sessions in male *Cacna1c*^+/^*^−^* rats (white circles; *N*=10) and *Cacna1c^+/+^* littermate controls (black circles; *N*=10). (D) 50-kHz USV emission during play versus non-play phases and (E) during individual play events in male *Cacna1c*^+/^*^−^* rats (white bars; *N*=10) and *Cacna1c^+/+^* littermate controls (black bars; *N*=10), with 50-kHz USVs being presented relative to the duration of play versus no-play phases and individual play events. (F) Representative, composite and consolidated ethograms of a *Cacna1c*^+/^*^−^* rat pair (upper panels) and a *Cacna1c^+/+^* littermate control pair (lower panels) of the first and third play session, respectively. Pinning (blue), wrestling (green) and chasing (brown) events are depicted, together with 50-kHz USVs (red) for the entire 5 min play sessions. Data are presented as mean±s.e.m. ^#^*P*<0.050 vs first play session (in A-C), vs play (in D), vs pinning (in E); ^##^*P*<0.050 vs pinning and wrestling (in E); *P<0.050 vs *Cacna1c^+/+^* littermate controls.

While *Cacna1c* haploinsufficiency did not lead to altered rough-and-tumble play behavior, concomitant emission of pro-social 50-kHz USVs was strongly affected. Specifically, there were no genotype differences in play behavior with regards to time spent playing [genotype (G): *F*_1,18_=0.037, *P*=0.849; Fig. 2A] or individual playful events, i.e. pinning (G: *F*_1,18_=0.045, *P*=0.835; Fig. 2B), wrestling (G: *F*_1,18_=0.046, *P*=0.833; Fig. S2A) and chasing (G: *F*_1,18_=1.333, *P*=0.263; Fig. S2B). Across play sessions, the time engaged in playful social interactions increased, regardless of genotype [day (D): *F*_2,36_=10.057, *P*<0.001; D×G: *F*_2,36_=0.246, *P*=0.783). This was driven by a genotype-independent increase in pinning and wrestling duration (D: *F*_2,36_=11.327, *P*<0.001; D×G: *F*_2,36_=0.171, *P*=0.844 and D: *F*_2,36_=10.748, *P*<0.001; D×G: *F*_2,36_=0.412, *P*=0.666, respectively), while chasing did not change (D: *F*_2,36_=0.671, *P*=0.518; D×G: *F*_2,36_=1.672, *P*=0.202).

Despite unchanged rough-and-tumble play behavior, however, *Cacna1c^+/−^* rats emitted fewer 50-kHz USVs than *Cacna1c^+/+^* littermates while engaged in playful encounters (G: *F*_1,17_=7.708, *P*=0.013; Fig. 2C). From the first play session, genotypes clearly differed, with prominent genotype effects being further evident during the second and third play session. During the anticipation phase, genotypes did not differ in 50-kHz USV emission (G: *F*_1,17_=1.537, *P*=0.232). Irrespective of genotype, there was an increase in 50-kHz USV emission during anticipation (D: *F*_2,35_=8.498, *P*=0.001; D×G: *F*_2,35_=1.057, *P*=0.359) and during playful social interactions (D: *F*_2,35_=20.901, *P*<0.001; D×G: *F*_2,35_=0.025, *P*=0.976) across play sessions.

When performing detailed temporal analyses in an additional exploratory approach, specifically for the third play session, genotype differences in 50-kHz USV emission were found to be robust (G: *F*_1,18_=16.159, *P*=0.009) and seen during play periods, i.e. while rats engaged in rough-and-tumble play behavior (*t*_18_=2.352, *P*=0.030), but also during non-play periods (*t*_18_=2.805, *P*=0.012; Fig. 2D). Within play periods, 50-kHz USV levels differed between individual rough-and-tumble play components [component (C): *F*_2,36_=16.159, *P*<0.001] and genotypes specifically during wrestling, with *Cacna1c^+/−^* rats emitting fewer 50-kHz USVs than *Cacna1c^+/+^* littermates (*t*_18_=2.529, *P*=0.021; Fig. 2E). No genotype effects were evident during the other two playful events, i.e. pinning (*t*_18_=0.290, *P*=0.775) and chasing (*t*_18_=0.395, *P*=0.697; for representative ethograms: Fig. 2F). In both *Cacna1c^+/−^* rats and *Cacna1c^+/+^* littermates, 50-kHz USV emission was higher during play than non-play periods (*t*_9_=3.021, *P*=0.014 and *t*_9_=3.180, *P*=0.011, respectively), with particularly high 50-kHz USV emission rates during wrestling and chasing but not pinning in *Cacna1c^+/+^* littermates (pinning versus wrestling: *t*_19_=3.783, *P*=0.004; pinning versus chasing: *t*_19_=4.529, *P*=0.001; wrestling versus chasing: *t*_19_=0.438, *P*=0.672), and during chasing but not pinning and wrestling in *Cacna1c^+/−^* rats (pinning versus wrestling: *t*_19_=2.124, *P*=0.063; pinning versus chasing: *t*_19_=3.737, *P*=0.005; wrestling versus chasing: *t*_19_=2.370, *P*=0.042).

**Fig. 3.**
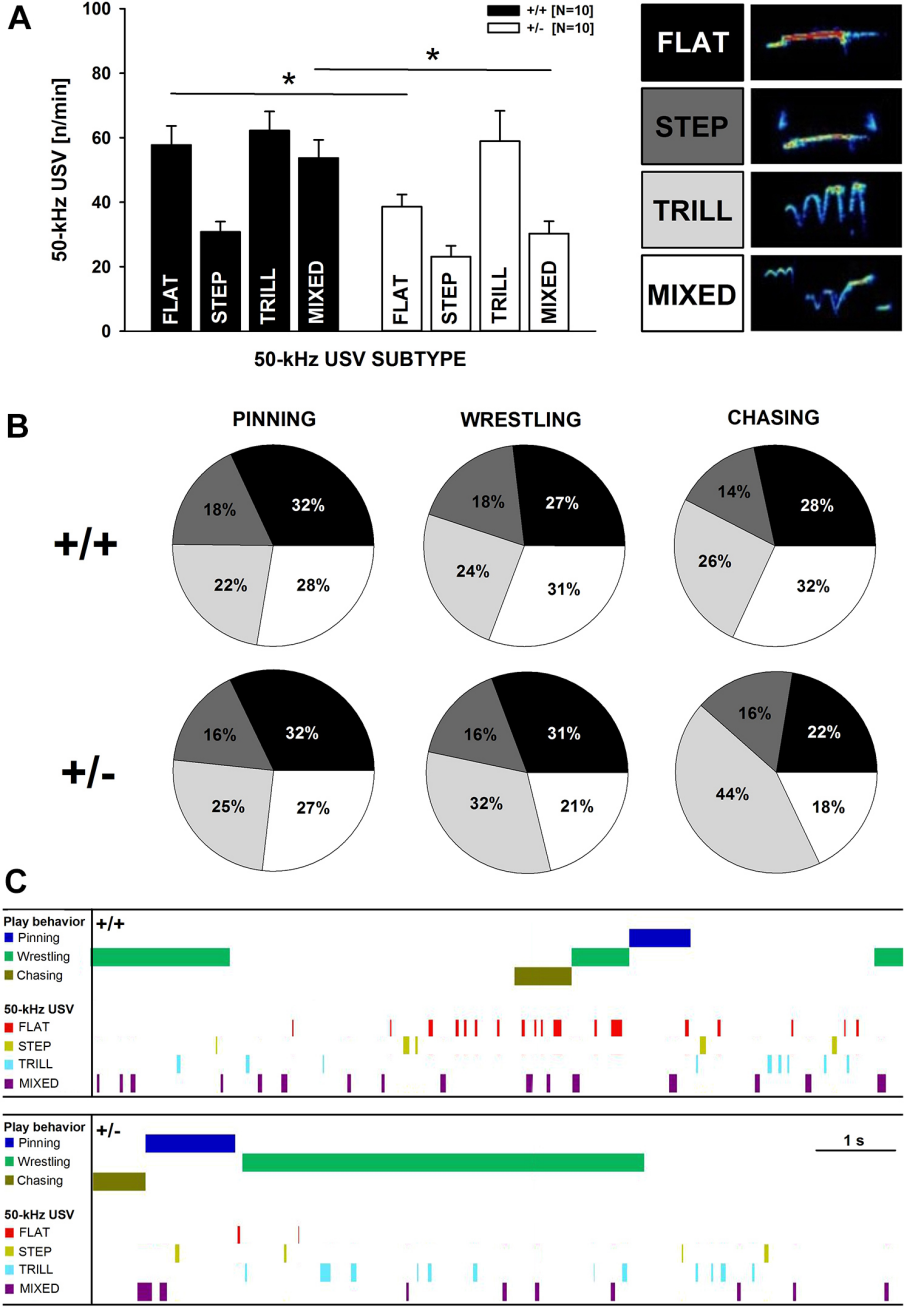
**Subtypes of pro-social 50-kHz USVs emitted by *Cacna1c*^+/^***^**−**^* **rats and *Cacna1c^+/+^* littermate controls during rough-and-tumble play behavior.** (A) Emission of the different 50-kHz USV subtypes, i.e. flat, step, trill and mixed 50-kHz USVs, in male *Cacna1c*^+/^*^−^* rats (white bars; *N*=10) and *Cacna1c^+/+^* littermate controls (black bars; *N*=10) during the third play session. (B) Pie charts depicting the proportion of the different 50-kHz USV subtypes emitted by male *Cacna1c*^+/^*^−^* rats (lower panel) and *Cacna1c^+/+^* littermate controls (upper panel) during individual play events, i.e. pinning, wrestling and chasing, during the third play session. The proportion of flat, step, trill and mixed 50-kHz USVs is shown in black, dark gray, light gray and white, respectively. (C) Detailed representative, composite and consolidated ethograms of a *Cacna1c*^+/^*^−^* rat pair (lower panel) and a *Cacna1c^+/+^* littermate control pair (upper panel) of the third play session. Pinning (blue), wrestling (green) and chasing (brown) events are depicted, together with the 50-kHz USV subtypes (modified to reflect order in text), i.e. flat (red), step (yellow), trill (turquoise) and mixed (purple), for 10 s of the entire 5 min play sessions. Data are presented as mean±s.e.m. *P<0.050 vs *Cacna1c^+/+^* littermate controls.

Moreover, differences in the prevalence of specific 50-kHz USV subtypes was evident [subtype (S): *F*_3,54_=16.696, *P*<0.001], with the genotype difference in 50-kHz USV emission rates being driven by reduced flat and mixed 50-kHz USVs [criteria previously established by Pereira et al. (2014) and repeatedly applied by Engelhardt et al. (2017) and Wöhr et al. (2015); Fig. 3A] in *Cacna1c^+/−^* rats, as compared to *Cacna1c^+/+^* littermates (*t*_18_=2.736, *P*=0.014 and *t*_18_=3.420, *P*=0.003, respectively). Step and trill 50-kHz USVs were not affected by genotype (*t*_18_=1.650, *P*=0.116 and *t*_18_=0.295, *P*=0.771, respectively; Fig. 3A). Importantly, genotype affected the 50-kHz USV profiles, i.e. the prevalence of specific 50-kHz USV subtypes, associated with individual rough-and-tumble play components (S: *F*_3,36_=6.570, *P*=0.001; S×G: *F*_3,36_=2.406, *P*=0.083; S×C: *F*_6,72_=3.545, *P*=0.004; S×C×G: *F*_6,72_=2.774, *P*=0.018; Fig. 3B; for representative ethograms: Fig. 3C). In *Cacna1c^+/+^* littermates, pinning was primarily associated with the occurrence of flat 50-kHz USVs and, to a lesser extent, mixed 50-kHz USVs, while trill and step 50-kHz USVs were rarely emitted. A similar pattern was obtained in *Cacna1c^+/−^* rats, with a large number of flat 50-kHz USVs, moderate levels of mixed and trill 50-kHz USVs, but low rates of step 50-kHz USVs. During wrestling, *Cacna1c^+/+^* littermates emitted high rates of mixed and flat 50-kHz USVs, together with moderate numbers of trill 50-kHz USVs but low numbers of step 50-kHz USVs. This was different in *Cacna1c^+/−^* rats, which produced a high number of trill and flat 50-kHz USVs during wrestling but relatively low numbers of mixed and particularly step 50-kHz USVs. During chasing, high levels of mixed 50-kHz USVs, moderate rates of flat and trill 50-kHz USVs, but low levels of step 50-kHz USVs were evident in *Cacna1c^+/+^* littermates. In *Cacna1c^+/−^* rats, trill 50-kHz USVs were most prominent, while flat, mixed and step 50-kHz USVs did not occur often during chasing. In rare cases, both rats were emitting 50-kHz USVs at the same time. The number of such overlapping 50-kHz USVs did not differ between genotypes (*t*_18_=1.472, *P*=0.158). Occasionally, atypical 50-kHz USVs were detected at comparable levels in both genotypes (*t*_18_=1.977, *P*=0.064).

**Fig. 4.**
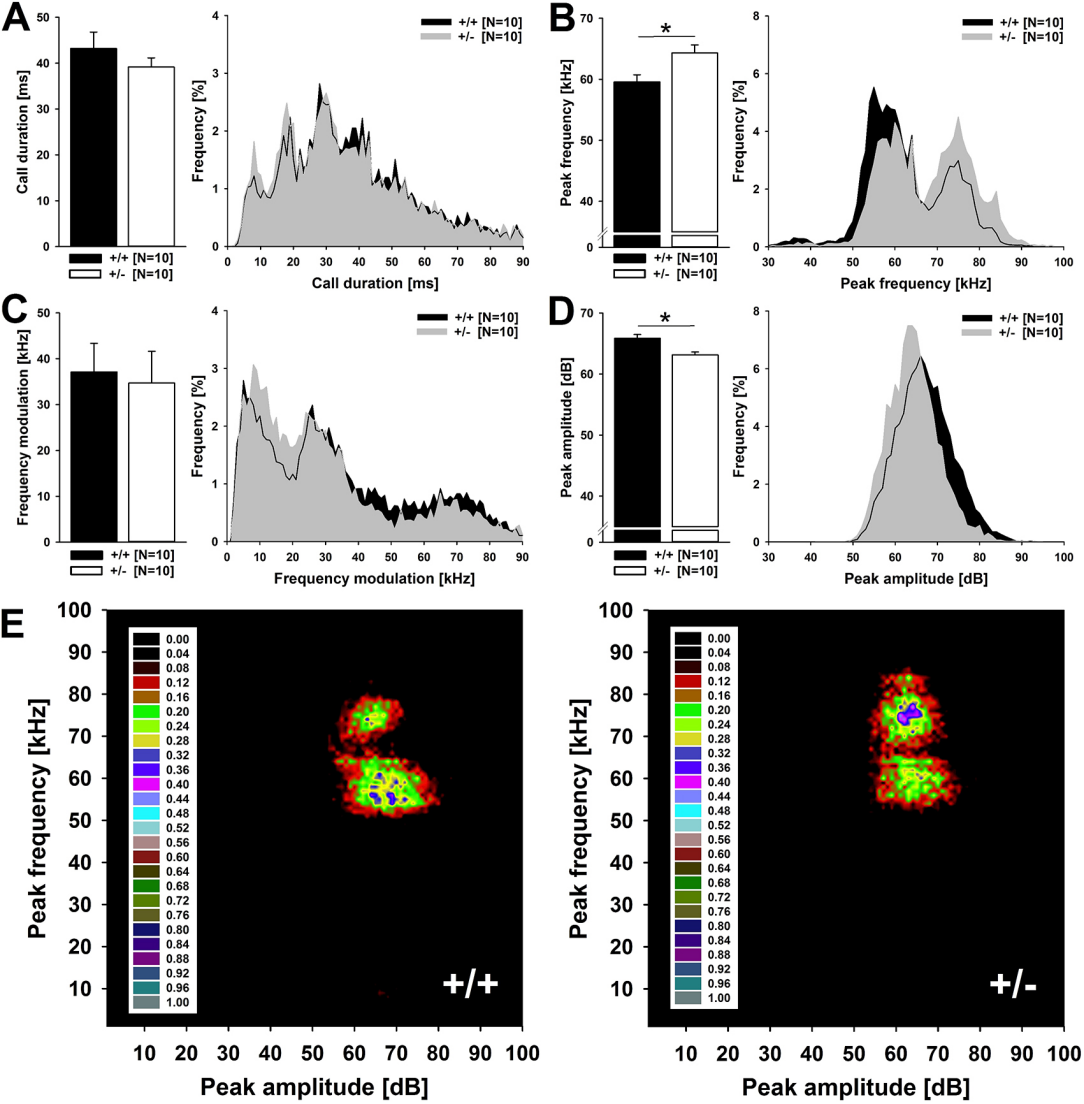
**Acoustic characteristics of pro-social 50-kHz USVs emitted by *Cacna1c*^+/^***^**−**^* **rats and *Cacna1c^+/+^* littermate controls during rough-and-tumble play behavior.** (A) Call duration [in milliseconds (ms)]; (B) peak frequency [in kilohertz (kHz); (C) frequency modulation (in kHz); and (D) peak amplitude [in decibel (dB)] of 50-kHz USVs emitted by male *Cacna1c*^+/^*^−^* rats (white bars and gray frequency histograms reflecting percentage of occurrence; *N*=10) and *Cacna1c^+/+^* littermate controls (black bars and black frequency histograms reflecting percentage of occurrence; *N*=10) during the third play session. (E) Density plots depicting the distribution of individual 50-kHz USVs depending on peak frequency (in kHz) and peak amplitude (in dB) emitted by male *Cacna1c*^+/^*^−^* rats (+/−; *N*=10) and *Cacna1c^+/+^* littermate controls (+/+; *N*=10) during the third play session. Color coding reflects frequencies of occurrence as percentages. Density plots were generated by including more than 8000 50-kHz USVs emitted by male *Cacna1c*^+/^*^−^* rats and more than 10,000 50-kHz USVs emitted by *Cacna1c^+/+^* littermate controls. Data are presented as mean±s.e.m. *P<0.050 vs *Cacna1c^+/+^* littermate controls.

Besides 50-kHz USV emission rates, acoustic characteristics of 50-kHz USVs differed between genotypes. While call duration was not affected (*t*_18_=0.987, *P*=0.337; Fig. 4A), 50-kHz USVs emitted by *Cacna1c^+/−^* rats were characterized by higher peak frequencies than the ones emitted by *Cacna1c^+/+^* littermates (*t*_18_=2.677, *P*=0.015; Fig. 4B), without differing in frequency modulation (*t*_18_=0.259, *P*=0.799; Fig. 4C). Moreover, 50-kHz USVs emitted by *Cacna1c^+/−^* rats were lower in peak amplitude (*t*_18_=3.330, *P*=0.004; Fig. 4D). The increase in peak frequency seen in *Cacna1c^+/−^* rats was driven by a categorical shift in the relative occurrence of 50-kHz USVs within two prominent call clusters. In both genotypes, two clusters were clearly evident. In the first cluster, 50-kHz USVs are characterized by relatively low peak frequencies, between 50 and 70 kHz. In the second cluster, 50-kHz USVs are characterized by substantially higher peak frequencies, between 70 and 90 kHz. *Cacna1c^+/+^* littermates emitted more low-frequency first-cluster 50-kHz USVs than high-frequency second-cluster 50-kHz USVs. Conversely, *Cacna1c^+/−^* rats emitted about the same number of first- and second-cluster 50-kHz USVs, resulting in an overall increase in peak frequency. In contrast to peak frequency, the decrease in peak amplitude seen in *Cacna1c^+/−^* rats was due to a gradual reduction (Fig. 4E).

### Playback of pro-social 50-kHz USVs

**Fig. 5.**
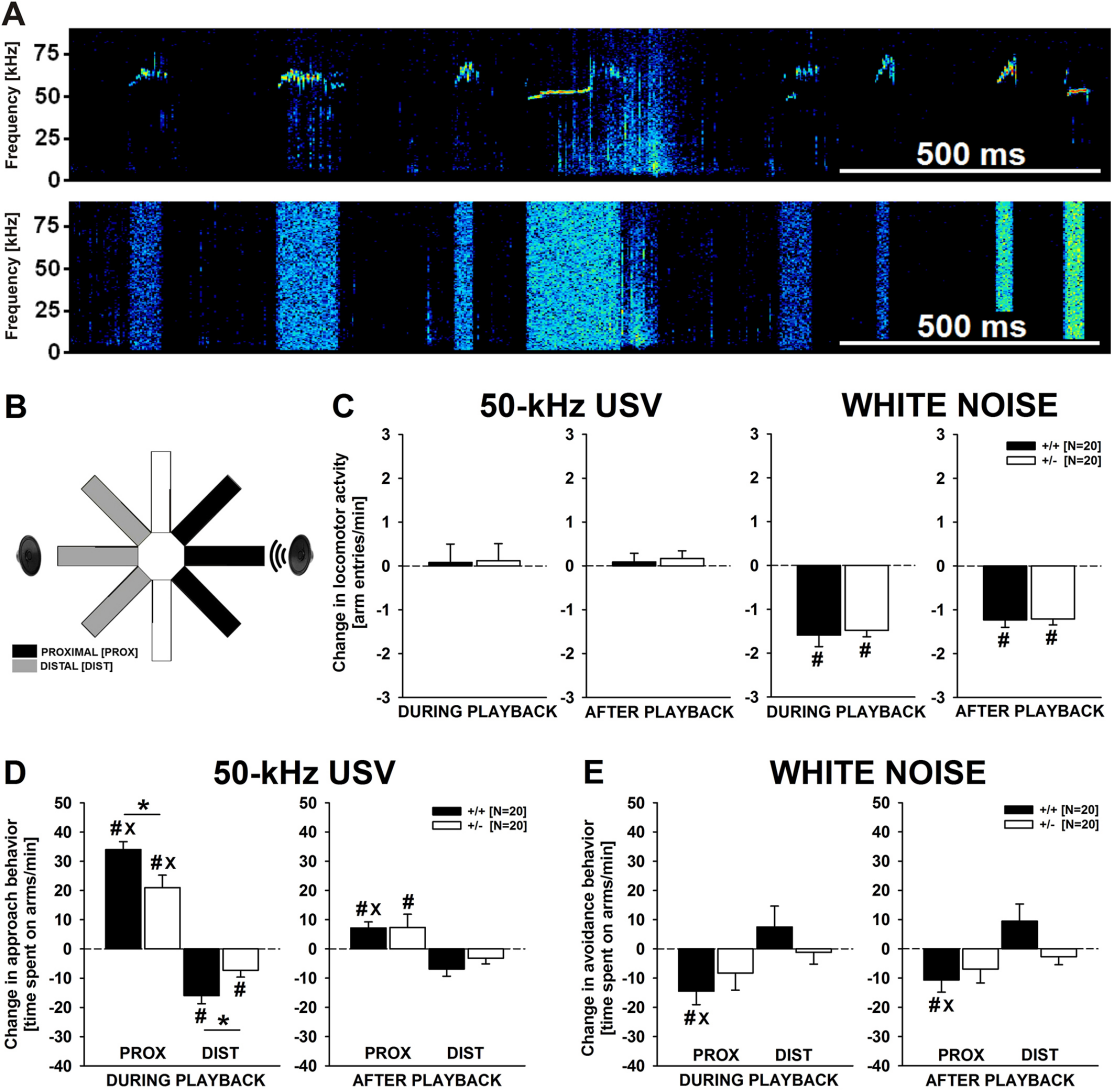
**Social approach behavior evoked by pro-social 50-kHz USV playback in *Cacna1c*^+/^*^−^*** **rats and *Cacna1c^+/+^* littermate controls.** (A) Exemplary spectrograms of acoustic stimuli used for playback, namely pro-social 50-kHz USVs (upper panel) and time- and amplitude-matched white noise (lower panel). (B) Schematic illustration of the radial maze used for playback depicting proximal (black), distal (gray) and neutral (white) arms relative to the active ultrasonic loudspeaker. (C) Change in locomotor activity in male *Cacna1c*^+/^*^−^* rats (white bars; *N*=20) and *Cacna1c^+/+^* littermate controls (black bars; *N*=20) as measured by total arm entries per minute during (left) and after (right) 50-kHz USV and white noise playback, as compared to the 5 min baseline period before playback. (D) Change in social approach behavior in male *Cacna1c*^+/^*^−^* rats (white bars; *N*=20) and *Cacna1c^+/+^* littermate controls (black bars; *N*=20) as measured by time spent on proximal (PROX) and distal (DIST) arms per minute during (left) and after (right) 50-kHz USV playback, as compared to the 5 min baseline period before playback. (E) Change in avoidance behavior in male *Cacna1c*^+/^*^−^* rats (white bars; *N*=20) and *Cacna1c^+/+^* littermate controls (black bars; *N*=20) as measured by time spent on proximal and distal arms per minute during (left) and after (right) white noise playback, as compared to the 5 min baseline period before playback. The dashed line represents baseline levels. Data are presented as mean±s.e.m. ^#^*P*<0.050 vs baseline levels; ^x^*P*<0.050 vs distal; *P<0.050 vs *Cacna1c^+/+^* littermate controls.

Importantly, low emission of pro-social 50-kHz USVs in the sender was paralleled by reduced responsivity to such 50-kHz USVs in the receiver, with 50-kHz USVs but not the acoustic control stimulus white noise (Fig. 5A) leading to social approach behavior, as demonstrated by means of our established 50-kHz USV radial maze playback paradigm (Fig. 5B). Specifically, the acoustic control stimulus white noise induced behavioral inhibition [time (T): *F*_1,38_=104.143, *P*<0.001; TxG: *F*_1,38_=0.134, *P*=0.717; Fig. 5C]. Both *Cacna1c^+/−^* rats and *Cacna1c^+/+^* littermates displayed reduced total arm entries during playback of white noise than before (T: *F*_1,19_=101.605, *P*<0.001 and *F*_1,19_=36.670, *P*<0.001, respectively). Moreover, behavioral inhibition was still evident after playback (T: *F*_1,38_=127.529, *P*<0.001; T×G: *F*_1,38_=0.009, *P*=0.927) and both genotypes continued to display reduced total arm entries after playback as compared to baseline (T: *F*_1,19_=80.422, *P*<0.001 and *F*_1,19_=52.123, *P*<0.001, respectively). No behavioral inhibition was seen in response to playback of 50-kHz USVs. As compared to baseline before playback, during and after playback there was no change in total arm entries, irrespective of genotype (T: *F*_1,38_=0.122, *P*=0.728; T×G: *F*_1,38_=0.005, *P*=0.945 and T: *F*_1,38_=0.977, *P*=0.329; T×G: *F*_1,38_=0.092, *P*=0.763, respectively). Of note, locomotor activity during the initial 15-min habituation period did not differ between genotypes, with total number of arm entries being similar in *Cacna1c^+/−^* rats and *Cacna1c^+/+^* littermates (G: *F*_1,38_=1.119, *P*=0.297; T×G: *F*_14,532_=1.270, *P*=0.222). Immediate head orientation in response to playback of 50-kHz USVs and white noise was seen in almost all rats (∼95%) and did not differ between genotypes (χ^2^=2.105, *P*=0.147). Not a single rat failed to respond to both acoustic stimuli by head orientation.

Social approach behavior in response to playback of 50-kHz USVs was reflected in a preference for arms proximal to the ultrasonic loudspeaker [T: *F*_1,38_=50.904, *P*<0.001; preference (P): *F*_1,38_=68.242, *P*<0.001; T×P: *F*_1,38_=103.775, *P*<0.001]. This preference was strongly dependent on genotype (T×G: *F*_1,38_=0.977, *P*=0.329; P×G: *F*_1,38_=1.292, *P*=0.263; T×P×G: *F*_1,38_=8.015, *P*=0.007; Fig. 5D). Although both *Cacna1c^+/−^* rats and *Cacna1c^+/+^* littermates displayed social approach behavior and spent more time proximal during playback than before (T: *F*_1,19_=23.608, *P*<0.001 and *F*_1,19_=155.747, *P*<0.001, respectively), but less time distal (T: *F*_1,19_=9.635, *P*=0.006 and *F*_1,19_=32.618, *P*<0.001, respectively), resulting in a preference for proximal over distal arms in both genotypes (P: *F*_1,19_=22.179, *P*<0.001 and *F*_1,19_=108.615, *P*<0.001, respectively), the strength of the response was clearly genotype dependent. In fact, the increase in time spent proximal was stronger in *Cacna1c^+/+^* than in *Cacna1c^+/−^* rats (*t*_38_=2.561, *P*=0.015). Likewise, the reduction in time spent distal was stronger in *Cacna1c^+/+^* littermates (*t*_38_=2.375, *P*=0.023). Similar genotype effects were evident in the minutes following 50-kHz USV playback (T: *F*_1,38_=0.766, *P*=0.387; T×G: *F*_1,38_=0.612, *P*=0.439; P: *F*_1,38_=19.212, *P*<0.001; P×G: *F*_1,38_=7.609, *P*=0.009; T×P: *F*_1,38_=13.409, *P*=0.001; T×P×G: *F*_1,38_=0.282, *P*=0.598). While *Cacna1c^+/+^* littermates continued displaying a preference for proximal over distal arms (P: *F*_1,19_=15.721, *P*=0.001), no clear preference was evident in *Cacna1c^+/−^* rats (P: *F*_1,19_=3.401, *P*=0.081). This was due to the fact that *Cacna1c^+/+^* littermates, but not *Cacna1c^+/−^* rats, kept spending more time proximal after playback than before (T: *F*_1,19_=11.799, *P*=0.003 and *F*_1,19_=2.607, *P*=0.123, respectively). They further kept spending less time distal (T: *F*_1,19_=7.797, *P*=0.012 and *F*_1,19_=2.635, *P*=0.121, respectively).

Besides the preference induced by 50-kHz USV playback, avoidance induced by the acoustic control stimulus white noise was modulated by genotype (T: *F*_1,38_=3.773, *P*=0.060; T×G: *F*_1,38_=0.085, *P*=0.772; P: *F*_1,38_=5.421, *P*=0.025; P×G: *F*_1,38_=11.467, *P*=0.002; T×P: *F*_1,38_=4.885, *P*=0.033; T×P×G: *F*_1,38_=1.289, *P*=0.263; Fig. 5E). In fact, *Cacna1c^+/+^* littermates displayed clear avoidance of proximal arms (P: *F*_1,19_=4.671, *P*=0.044), with the time spent on proximal arms being reduced during as compared to before playback (T: *F*_1,19_=9.922, *P*=0.005) and the time spent on distal arms being unchanged (T: *F*_1,19_=1.103, *P*=0.307). No such avoidance of proximal arms was evident in *Cacna1c^+/−^* rats (P: *F*_1,19_=0.721, *P*=0.406), with the time spent on proximal and distal arms being unchanged (T: *F*_1,19_=1.996, *P*=0.174 and *F*_1,19_=0.090, *P*=0.767, respectively). A similar pattern was evident following white noise playback (T: *F*_1,38_=2.776, *P*=0.104; T×G: *F*_1,38_=1.672, *P*=0.204; P: *F*_1,38_=8.358, *P*=0.006; P×G: *F*_1,38_=13.943, *P*=0.001; T×P: *F*_1,38_=4.959, *P*=0.032; T×P×G: *F*_1,38_=2.106, *P*=0.155). Again, *Cacna1c^+/+^* littermate controls displayed clear avoidance of proximal arms (P: *F*_1,19_=4.997, *P*=0.038), with reduced time spent on proximal arms (T: *F*_1,19_=6.607, *P*=0.019) and unchanged time spent on distal arms (T: *F*_1,19_=2.628, *P*=0.121). No avoidance was evident in *Cacna1c^+/−^* rats (P: *F*_1,19_=0.465, *P*=0.503), with the time spent on proximal and distal arms being unchanged (T: *F*_1,19_=2.152, *P*=0.159 and *F*_1,19_=0.976, *P*=0.336, respectively).

### Repetitive and stereotyped patterns of behavior

Finally, *Cacna1c* haploinsufficiency did not lead to enhanced levels of repetitive and stereotyped patterns of behavior, with tail chasing (*t*_38_=0.211, *P*=0.834; Fig. S3A) and self-grooming (*t*_38_=1.127, *P*=0.267; Fig. S3B) occurring at similar levels in both genotypes. Of note, locomotor activity during the assessment of repetitive and stereotyped patterns of behavior was not affected by genotype. Specifically, line crossings (*t*_38_=1.538, *P*=0.132) and rearing events (*t*_38_=1.517, *P*=0.137) occurred at similar levels in *Cacna1c^+/−^* rats and *Cacna1c^+/+^* littermates.

## DISCUSSION

*CACNA1C* has emerged as a prime candidate susceptibility gene for neuropsychiatric disorders, particularly because single-nucleotide polymorphisms (SNPs) in *CACNA1C* rank among the most consistent and replicable findings from genome-wide association studies (Cross-Disorder Group of the Psychiatric Genomics Consortium, 2013). However, as rs1006737 and other identified SNPs are found in the intronic, i.e. the non-protein-coding, region of *CACNA1C*, neurobiological mechanisms whereby such SNPs modify brain structure and function are not well understood. In fact, some reports have associated the risk variant rs1006737 with enhanced *CACNA1C* mRNA expression in post-mortem tissue and induced human neurons (Yoshimizu et al., 2015), whereas others reported decreased *CACNA1C* expression levels in the brains of SCZ and BPD patients carrying this risk allele (Gershon et al., 2014). LTCC activity is also perturbed in a rare yet devastating disorder known as Timothy syndrome (TS) with features partly similar to ASD. Most cases arise from a G406R *CACNA1C* missense mutation (Splawski et al., 2004) and a TS mouse model carrying the G406R replacement in Ca_v_1.2 was reported to display ASD-related behavioral phenotypes (Bader et al., 2011). To our knowledge, however, behavioral phenotypes with relevance for socio-affective communication deficits in ASD, BPD and SCZ have not been assessed in rats with genetic modifications targeting *Cacna1c* until now, and available mouse studies almost exclusively focused on adult mice (Kabir et al., 2016), with no data being available on the role of *Cacna1c* in regulating socio-affective communication during the critical developmental period of adolescence.

Our results show for the first time that *Cacna1c* deletion leads to pro-social 50-kHz ultrasonic communication deficits and may suggest reduced incentive salience of social contact in *Cacna1c* haploinsufficient rats. While *Cacna1c* haploinsufficiency did not lead to altered rough-and-tumble play behavior, concomitant emission of 50-kHz USVs was strongly affected. Over all three play sessions, *Cacna1c^+/−^* rats consistently emitted fewer 50-kHz USVs while engaged in playful social interactions than *Cacna1c^+/+^* littermate controls. Genotype differences were evident during play and non-play periods, with *Cacna1c^+/−^* rats only reaching non-play period 50-kHz USV levels of *Cacna1c^+/+^* littermate controls during play periods. In an initial effort to link 50-kHz USV emission to specific individual playful events, we additionally showed, for the first time, by means of temporal analyses using high-resolution ethograms, that wrestling and chasing are associated with particularly high 50-kHz USV rates in *Cacna1c^+/+^* littermate controls. Notably, this association was mild in *Cacna1c^+/−^* rats and low rates of 50-kHz USVs were detected during wrestling. Within play periods, the genotype difference in 50-kHz USVs was thus driven by reduced emission rates during wrestling but not pinning or chasing. When performing a detailed spectrographic analysis, we further found that *Cacna1c* haploinsufficiency affected the 50-kHz USV profile by reducing flat and mixed 50-kHz USV subtypes previously associated with the synchronization of complex social interactions (Łopuch and Popik, 2011). Particularly during chasing, the prevalence of trill 50-kHz USVs was enhanced in *Cacna1c^+/−^* rats at the expense of mixed 50-kHz USVs. Moreover, acoustic characteristics were found to be altered, with peak frequency being higher but peak amplitude being lower in *Cacna1c^+/−^* rats. This was at least in part due to alternative clustering. Together, since 50-kHz USVs are believed to reflect positive affective states (‘rat laughter’) (Panksepp, 2005) associated with the rewarding nature of rough-and-tumble play (Vanderschuren et al., 2016), this suggests that *Cacna1c^+/−^* rats derive lower levels of reward from playful encounters, possibly due to impaired ‘liking’ (Berridge et al., 2009).

Besides the emission of fewer 50-kHz USVs in the sender, *Cacna1c* deletion reduced the behavioral responses elicited by 50-kHz USV playback, with social approach behavior clearly being more prominent in *Cacna1c^+/+^* littermate controls than in *Cacna1c^+/−^* rats. Importantly, genotype differences are unlikely due to auditory processing deficits. Immediate head orientation in response to playback of 50-kHz USVs or white noise was seen in all rats and did not differ between genotypes. Moreover, both genotypes displayed behavioral inhibition when exposed to white noise playback, with the strength of the response not differing between genotypes. However, *Cacna1c^+/+^* littermate controls, but not *Cacna1c^+/−^* rats, further displayed clear avoidance behavior and moved away from the sound source in response to white noise playback. The avoidance response displayed by *Cacna1c^+/+^* littermate controls was long-lasting and still seen in the minutes following playback. Lack of avoidance in *Cacna1c^+/−^* rats might appear surprising given the ample evidence for increased anxiety-related behavior in constitutive *Cacna1c* heterozygous mice (Lee et al., 2012), particularly in females (Dao et al., 2010), yet strong behavioral inhibition seen in both genotypes speaks for alterations in coping strategies rather than anxiety levels. Finally, genotype differences in social approach behavior in response to 50-kHz USV playback were not due to impairments in behavioral activity and motor functions. Locomotor activity and rearing behavior did not differ between genotypes. Together, this suggests that genotype differences in social approach behavior evoked by 50-kHz USVs reflects genotype effects on the motivation, i.e. ‘wanting’, for social contact, which is expressed in the amount of effort spent to obtain a social reward (Berridge et al., 2009). Notably, the observed deficits in social approach behavior in response to 50-kHz USVs are more prominent in our newly developed rat model than in a well-established *Shank3* rat model for ASD (Berg et al., 2018), emphasizing the severity of the social deficits displayed by *Cacna1c* haploinsufficient rats. Together with the reduced 50-kHz USV emission rates during playful social interactions, this may, therefore, suggest deficits in ‘wanting’ in addition to the ‘liking’ component associated with playful encounters. Interestingly, reward processing and 50-kHz ultrasonic communication are both linked to dopamine (Burgdorf et al., 2007). Thus, 50-kHz USV playback evokes phasic dopamine release in the nucleus accumbens (Willuhn et al., 2014) and dopamine signaling is profoundly altered in genetic *Cacna1c* mouse models (Terrillion et al., 2017a).

Our results indicate that a deletion of *Cacna1c* leads to deficits in social behavior and pro-social 50-kHz ultrasonic communication in rats. This is at least partially in line with currently available mouse studies. Traditionally, social behavior in mouse models is assessed using the three-chambered social approach assay, with intact sociability being defined as spending more time in proximity to a conspecific over an empty corral (Silverman et al., 2010). Using this classic assay, Kabir et al. (2017) and Dedic et al. (2018) found that adult forebrain *Cacna1c-*null mutant mice do not show a preference for the conspecific. Lack of sociability was also seen after *Cacna1c* knockdown specifically in the prefrontal cortex (Kabir et al., 2017), but not the nucleus accumbens (Terrillion et al., 2017b). Moreover, in a modified version of the task, a mild reduction in sociability was seen in the TS mouse model carrying the G406R replacement in Ca_v_1.2 (Bader et al., 2011; but see Kabitzke et al., 2018), although this is a gain-of-function mutation in Ca_v_1.2 characterized by reduced inactivation (Splawski et al., 2004). Further evidence for a role of *Cacna1c* in regulating socio-affective communication comes from a study by Jeon et al. (2010), who showed that observational fear learning in mice is impaired following local Ca_v_1.2 deletion in the anterior cingulate cortex. However, in constitutive *Cacna1c* heterozygous mice, no evidence for social deficits was obtained in two independent studies (Bader et al., 2011; Dedic et al., 2018) (for a comprehensive overview on the behavioral effects of genetic modifications targeting *Cacna1c* in mice, see Kabir et al., 2016). The fact that social deficits were only evident in *Cacna1c* null mutant but not *Cacna1c* heterozygous mice, although, in rats, *Cacna1c* haploinsufficiency already results in deficits, is possibly due to the richer social behavior repertoire of rats, with pro-social 50-kHz USVs being particularly sensitive for detecting disorder-relevant behavioral phenotypes.

In summary, reduced levels of 50-kHz USVs emitted during rough-and-tumble play may suggest that *Cacna1c* haploinsufficient rats derive less reward from playful social interactions. Besides the emission of fewer 50-kHz USVs in the sender, *Cacna1c* deletion reduced the behavioral responses elicited by 50-kHz USV playback. This indicates that *Cacna1c* haploinsufficiency has detrimental effects on 50-kHz ultrasonic communication in both sender and receiver. Together, these data suggest that *Cacna1c* plays a prominent role in regulating socio-affective communication in rats with relevance for ASD, BPD and SCZ.

## MATERIALS AND METHODS

### Ethics approval

All procedures were conducted in strict accordance with the National Institutes of Health Guidelines for the Care and Use of Laboratory Animals and the relevant local or national rules and regulations of Germany, and were subject to prior authorization by the local government (MR 20/35 Nr. 19/2014; Tierschutzbehörde, Regierungspräsdium Gieβen, Germany).

### Animals and housing

Effects of *Cacna1c* haploinsufficiency on behavioral phenotypes with relevance for socio-affective communication deficits in ASD, BPD and SCZ were assessed in male constitutive heterozygous *Cacna1c^+/−^* rats (*N*=20) and compared to wild-type *Cacna1c^+/+^* littermate controls (*N*=20). *Cacna1c^+/−^* rats were generated by means of zinc-finger technology by SAGE Labs (now Horizon Discovery Ltd, Cambridge, UK) on a Sprague-Dawley (SD) background, following a previously established protocol (Geurts et al., 2009). *Cacna1c^+/−^* rats carry a 4 base pair (bp) deletion at 460,649-460,652 bp in the genomic sequence, resulting in an early stop codon in exon 6. Homozygous *Cacna1c^−/−^* rats are embryonically lethal.

A heterozygous breeding protocol was used to obtain offspring from both genotypes. To this aim, SD females (Charles River, Sulzfeld, Germany) and male *Cacna1c^+/−^* rats were paired for breeding. SD females were used because breeding efficacy is reduced in female *Cacna1c^+/−^* rats. *N*=8 litters with *N*=16.25±0.67 pups were obtained, with equal sex (*t*_7_=0.143, *P*=0.809) and genotype (*t*_7_=0.540, *P*=0.606) ratios. In order to avoid litter effects, only litters with both genotypes present were included in the experiments. Breeding was performed at the Faculty of Psychology, Philipps University of Marburg, Germany. Approximately 2 weeks after pairing for breeding, females were individually housed and inspected daily for pregnancy and delivery. The day of birth was considered as postnatal day (PND) 0. After weaning on PND 21, rats were socially housed in groups of 4-6 with same-sex littermate partners in polycarbonate Macrolon Type IV cages (Tecniplast Deutschland GmbH, Hohenpeiβenberg, Germany; 58×38×20 cm, length×width×height) under standard laboratory conditions (22±2°C and 40-70% humidity) with free access to standard rodent chow and water. Rats were identified by paw tattoo, applied using a non-toxic animal tattoo ink (Ketchum permanent tattoo inks green paste, Ketchum Manufacturing Inc., Brockville, Canada). The ink was inserted subcutaneously through a 30-gauge hypodermic needle tip into the center of the paw on PND 5±1.

### Genotyping

Rat tail snips were collected by dissecting ∼0.3 cm of tail on PND 5±1. Tails were digested, genomic DNA was isolated and purified using the Qiagen DNAeasy Blood and Tissue Kit according to the manufacturer's instructions (Hilden, Germany). After the extraction, 2.0 μl of DNA in buffer containing ∼250-400 μg of DNA was amplified by PCR using the Promega PCR Master Mix (Mannheim, Germany). The following primers were used: 5′-GCTGCTGAGCCTTTTATTGG-3′ (*Cacna1c* Cel-1 F) and 5′-CCTCCTGGATAGCTGCTGAC-3′ (*Cacna1c* Cel-1 R). Genotyping was performed on a 3130xl Genetic Analyzer (Thermo Fisher Scientific, Waltham, MA, USA).

### Protein analysis

Protein extraction and western blot were performed using frozen cortical tissue pieces (25-50 mg, left hemisphere) from 10-month-old male *Cacna1c^+/−^* rats (*N*=6) and their *Cacna1c^+/+^* littermate controls (*N*=6). Each tissue sample was lysed in 600 µl buffer containing 50 mM Tris hydrochloride, 150 mM sodium chloride, 5 mM EDTA, 1% (w/v) Triton X-100 and 0.5% (w/v) sodium deoxycholate supplemented with protease and phosphatase inhibitor cocktail tablets (Roche Diagnostics, Mannheim, Germany) and homogenized with T10 basic Ultra-Turrax (IKA-Werke, Staufen, Germany) for 10 s. The homogenates were then centrifuged for 15 min at 13,000 ***g*** at 4°C (Heraeus Fresco^TM^ 17, Thermo Fisher Scientific, Darmstadt, Germany). The total protein amount was determined from the supernatants using the Pierce BCA Protein Assay Kit (Thermo Fisher Scientific, Darmstadt, Germany). A total of 50 µg protein per sample were loaded on a 7.5% polyacrylamide gel. After electrophoresis, the proteins were transferred onto a PVDF membrane (Roche Diagnostics, Mannheim, Germany) and incubated with anti-Ca_v_1.2 (1:500; Cat# ACC-003; Lot# ACC003AN5102; Alomone Labs, Jerusalem, Israel) and anti-Vinculin antibodies (1:20,000; Sigma-Aldrich, München, Germany) overnight at 4°C. Protein detection was realized using peroxidase-labeled secondary antibodies (Vector Laboratories, Burlingame, CA, USA) and luminol-based HRP-Juice Plus (PJK GmbH, Kleinblittersdorf, Germany). The resulting chemiluminescence was imaged with a ChemiDoc XRS system (Bio-Rad Laboratories, Hercules, CA, USA). Protein quantification was performed using Bio-Rad Image Lab^TM^ Software. Unless otherwise stated, all reagents were purchased from Sigma-Aldrich (München, Germany).

### Behavioral phenotyping

Behavioral phenotypes were assessed in male *Cacna1c^+/−^* rats and compared to *Cacna1c^+/+^* littermate controls by means of our established 50-kHz USV radial maze playback paradigm (PND 24±3), rough-and-tumble play behavior and pro-social 50-kHz USVs (PND 32-34), as well as repetitive and stereotyped patterns of behavior (PND 64±3). All rats were tested in all three behavioral assays. Body weight did not differ between genotypes (for details, see Table S1; *t*_38_=0.859, *P*=0.396; *t*_18_=0.347, *P*=0.732 and *t*_38_=0.166, *P*=0.869, respectively), in line with a lack of body weight differences and genotype effects on general health measures during early development, as assessed in an independent cohort of rats to avoid potential confounds due to repeated handling. Behavioral experiments were carried out during the light phase of a 12:12 h light/dark cycle (lights on at 06:00 h). Rats were handled for three consecutive days prior to behavioral testing in a standardized way for 5 min. Behavioral analysis was performed by an experienced observer blind to experimental condition.

### Rough-and-tumble play and pro-social 50-kHz USVs

On PND 32-34, rough-and-tumble play behavior and the emission of pro-social 50-kHz USVs were measured, using sample sizes and a modified protocol previously established (Lukas and Wöhr, 2015). In rats, rough-and-tumble play behavior peaks around the age of PND 30-40 (Panksepp, 1981). On three consecutive days, pairs of juvenile rats were allowed to socially interact for 5 min (referred to as the play phase) in an, at first, unfamiliar observation arena (35×35 cm, with Plexiglas walls; floor covered with 1 cm of fresh bedding) after one rat of the pair had been habituated to the test environment for 2 min (referred to as the anticipation phase). A 3 day protocol was applied in order to assess changes in rough-and-tumble play and 50-kHz USV emission induced by play experience, such as anticipatory 50-kHz USVs (Knutson et al., 1998). Rats were always paired with a same-sex, same-genotype, age-matched non-littermate and non-cagemate partner, since it is not yet possible to identify the sender of pro-social 50-kHz USVs during rough-and-tumble play behavior in a reliable manner. To enhance the level of social motivation, subject rats were socially isolated for 24 h prior to testing in a Makrolon type III cage (265×150×425 mm, plus high stainless-steel covers; Tecniplast Deutschland GmbH), and isolation was maintained throughout the 3-day testing period. For behavioral analyses, a digital camera (TK-1281 Color Video Camera, JVC, Yokohama, Japan) was used and connected to an external multimedia hard drive (ScreenPlay Pro HD, Iomega, San Diego, CA, USA). The following behavioral measures were scored by an experienced observer using The Observer XT (Noldus, Wagenigen, The Netherlands): duration of rough-and-tumble play, including pinning, wrestling and chasing. Pinning was defined as one rat lying with its dorsal surface on the floor with the other rat standing over it. Wrestling was scored when a group of play-specific behaviors, including wrestling, boxing and pouncing, occurred. Chasing was defined as moving in the direction of or pursuing the partner while the partner is moving away. Pro-social 50-kHz USVs were recorded using an UltraSoundGate Condenser Microphone (CM16; Avisoft Bioacoustics, Berlin, Germany) placed 35 cm above the floor of the center of the observation arena. In an additional exploratory approach, detailed temporal analyses for linking individual playful events and 50-kHz USVs were performed for the third play session by means of high-resolution ethograms using The Observer XT. The generated composite ethograms representative for the first and third play session, respectively, were modified using a free and open source image editor, GIMP, with time reference, genotype and play session being manually added. Notably, a red relative-time indicator used by The Observer XT and subsequently copied into the image export was removed, as it noticeably obscured data presentation. Rough-and-tumble play behavior and the emission of pro-social 50-kHz USVs were measured under red light (∼28 lux).

### Playback of pro-social 50-kHz USVs

On PND 24±3, social approach behavior in response to pro-social 50-kHz USVs was assessed on an elevated radial eight-arm maze (arms: 40.5×9.8 cm) under red light (∼10 lux) according to a modified playback protocol previously established (Seffer et al., 2015). Particularly in males, social approach behavior induced by pro-social 50-kHz USVs is clearly more prominent in juvenile than adult rats (Wöhr and Schwarting, 2007). Acoustic stimuli were presented through an ultrasonic loudspeaker (ScanSpeak, Avisoft Bioacoustics) placed 20 cm away from the end of one arm. An additional, but inactive, loudspeaker was arranged symmetrically at the opposite arm as a visual control. Two acoustic stimuli were used: (1) pro-social 50-kHz USVs and (2) white noise; the latter serving as a time- and amplitude-matched acoustic stimulus control (Seffer et al., 2014). Pro-social 50-kHz USVs used for playback were recorded from a male rat during exploration of a cage containing scents from a recently separated cage mate. After an initial 15 min habituation period, each subject rat was exposed to 1 min playback presentations of 50-kHz USVs and white noise, separated by a 10 min inter-stimulus interval. Stimulus order was counterbalanced to account for possible sequence effects. The session ended after an additional 10 min post-stimulus phase. Behavior was monitored by a video camera (Panasonic WV-BP 330/GE, Hamburg, Germany) mounted centrally above the arena. In response to 50-kHz USV and white noise playback, immediate head orientation was quantified. Total number of arm entries served as a measure for locomotor activity. Change values were calculated by subtracting the total number of arm entries per minute during the 5 min baseline period before playback from the total number of arm entries per minute during and after 50-kHz USV and white noise playback, respectively. Time spent on arms proximal and distal to the active ultrasonic loudspeaker was used to quantify approach and avoidance behavior, respectively. Change values were calculated by subtracting the time spent on proximal and distal arms per minute during the 5 min baseline period before playback from the time spent on proximal and distal arms per minute during and after 50-kHz USV playback. USVs were monitored with two ultrasonic condenser microphones (CM16, Avisoft Bioacoustics) placed next to the loudspeakers.

### Recording and analysis of USVs

UltraSoundGate Condenser CM16 Microphones (Avisoft Bioacoustics) sensitive to frequencies of 15-180 kHz (flat frequency response between 25 and 140 kHz; ±6 dB) were used for USV recordings. They were connected via an UltraSoundGate 416H USB audio device (Avisoft Bioacoustics) to a personal computer, where acoustic data were recorded with a sampling rate of 250,000 Hz in 16-bit format (recording range: 0-125 kHz) by Avisoft RECORDER USGH. For acoustical analysis, recordings were transferred to Avisoft SASLab Pro (version 4.50). High-resolution spectrograms (frequency resolution: 488 Hz; time resolution: 0.512 ms) were obtained through a fast Fourier transformation (512 FFT length, 100% frame, Hamming window and 75% time window overlap). Call detection of pro-social 50-kHz USVs emitted by juvenile rats during rough-and-tumble play was provided by an experienced observer, who manually counted the number of USVs in 20 s time bins. If two 50-kHz USV elements were at least 10 ms apart, two independent 50-kHz USVs were counted. Based on previous studies on 50-kHz USVs, additional parameters were determined for ∼20,000 50-kHz USVs emitted during the third play session, including call duration, peak frequency, frequency modulation and peak amplitude (Wöhr et al., 2015). Peak frequency and peak amplitude were derived from the average spectrum of the entire call. The extent of frequency modulation was defined as the difference between the lowest and the highest peak frequency within each call. Moreover, the 50-kHz USV profile was determined and 50-kHz USVs emitted during the third play session were categorized into flat, step, trill and mixed 50-kHz USV subtypes using previously established (Pereira et al., 2014) and repeatedly applied (Engelhardt et al., 2017; Wöhr et al., 2015) criteria. Only rats emitting more than five calls per individual rough-and-tumble play component were included when comparing the prevalence of specific 50-kHz USV subtypes as percentages. In addition, the occurrence of atypical 50-kHz USVs with comparatively low peak frequencies below 32 kHz and/or long call durations higher than 150 ms was determined. Finally, overlapping 50-kHz USVs, i.e. when both rats were emitting 50-kHz USVs at the same time, were included in the detailed spectrographic analysis. One subject rat was excluded from the analysis of USVs of the first play session due to data loss.

### Repetitive and stereotyped patterns of behavior

On PND 64±3, repetitive and stereotyped patterns of behavior were tested in a clean Makrolon type III cage (265×150×425 mm, plus high stainless-steel covers; Tecniplast Deutschland GmbH) without bedding material. For behavioral analyses, a digital camera (TK-1281 Color Video Camera, JVC) was used and connected to an external multimedia hard drive (ScreenPlay Pro HD, Iomega). Repetitive and stereotyped patterns of behavior were assessed by measuring the duration of self-grooming and circling behavior during tail chasing. For assessing locomotor activity, the test cage was virtually divided in two halves by a line and the numbers of line crossings and rearing events were counted. Testing was performed under white light (∼30 lux) conditions for 20 min.

### Statistical analysis

For comparing rough-and-tumble play behavior and pro-social 50-kHz USVs between genotypes, analysis of variances (ANOVAs) for repeated measurements were calculated with the between-subject factor genotype (G) and the within-subject factors day (D), individual rough-and-tumble play components (C) and prevalence of specific 50-kHz USV subtypes (S), i.e. 50-kHz USV profiles. Playback of pro-social 50-kHz USVs was analyzed using ANOVAs for repeated measurements with the between-subject factor genotype (G) and the within-subject factors time (T) and preference (P). Acoustic characteristics of 50-kHz USVs, repetitive and stereotyped patterns of behavior, line crossings and rearing events, and Ca_v_1.2 protein levels were compared between genotypes by means of unpaired *t*-tests. The χ^2^-test was applied to compare immediate head orientation between genotypes. A *P*-value of <0.050 was considered statistically significant.

## Supplementary Material

Supplementary information

First Person interview
