# Supplementary material for: Cacna1c haploinsufficiency leads to pro-social 50-kHz ultrasonic communication deficits in rats
Source: Dis Model Mech. 2018 Jun 20;11(6):dmm034116. doi: 10.1242/dmm.034116 (PMC6031367; doi:10.1242/dmm.034116)
Supplement: Supplementary information [file dmm-11-034116-s1.pdf]

FIRST PERSON

First person – Theresa Kisko

First Person is a series of interviews with the first authors of a selection of papers published in Disease Models & Mechanisms, helping early-career researchers promote themselves alongside their papers. Theresa Kisko is first author on '*Cacna1c* haploinsufficiency leads to pro-social 50-kHz ultrasonic communication deficits in rats', published in DMM. Theresa is a PhD student in the lab of Markus Wöhr at Philipps-University of Marburg, Germany, investigating the development of pro-social behavior and communication with relevance to mood and neurodevelopmental disorders in rodents.

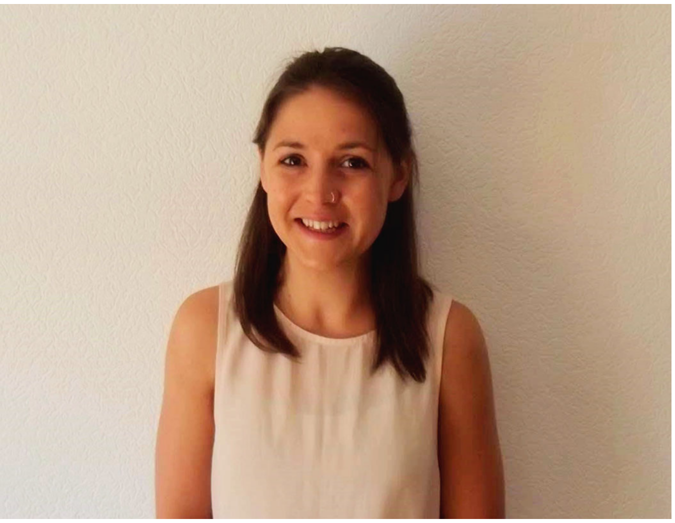

Theresa Kisko

How would you explain the main findings of your paper to non-scientific family and friends?

Across the world, individuals suffering with mood and neurodevelopmental disorders struggle with abnormal social behavior and communication impairments, contributing to an overall decrease in their quality of life. Recently, a gene known as *CACNA1C* has been associated with several mood and neurodevelopmental disorders in humans and, similarly, linked to associated impairments in rodent models. By using a newly developed *Cacna1c* rat model with only one intact copy of the gene, we explored the role *Cacna1c* has on the development of social behavior and communication, and its relevance to impairments seen in mood and neurodevelopmental disorders. We chose rats because they are a very social species and engage in high levels of social play as juveniles. When playing, but also when being tickled by a human hand, they emit ultrasonic vocalizations (USV) not audible to us humans because of their very high frequency around 50 kHz and thus clearly above the human hearing threshold of 20 kHz. These so-called 50-kHz USV are thought to reflect a positive effect akin to human laughter and serve an important communicative function as social contact calls. In detail, we investigated social play behavior, the production of 50-kHz USV, and the behavioral response to 50-kHz USV playback. Our results have now provided the first evidence implicating *Cacna1c* in social communication deficits in both the production of and response to emotionally relevant stimuli. That is, the model rats displayed deficits both as sender and receiver of the signals.

What are the potential implications of these results for your field of research?

Applying an ethologically valid approach, we have provided evidence that alterations in the *Cacna1c* gene in juvenile male rats leads to diminished emission of 50-kHz USV during social play and impaired social approach behavior when presented with playback of 50-kHz USV. Our results suggest that the emotional incentive salience, otherwise known as the 'wanting' or 'desire' for the pro-social interactions, may be impaired. Social withdrawal and amotivation are common characteristics in people suffering from mood and neurodevelopmental disorders.

Theresa Kisko's contact details: Behavioral Neuroscience, Experimental and Biological Psychology, Faculty of Psychology, Philipps-University of Marburg, D-35032 Marburg, Germany.

E-mail: kiskot@staff.uni-marburg.de

"Rats are an ideal model when studying social behavior because similar to humans, their social systems, especially at an early age, are integral to normal development."

What are the main advantages and drawbacks of the model system you have used as it relates to the disease you are investigating?

The *CACNA1C* gene in humans is associated with several mood and neurodevelopmental disorders in which social impairments are prominent characteristics. Rats are an ideal model when studying social behavior because similar to humans, their social systems, especially at an early age, are integral to normal development. Moreover, they also often engage in pro-social helping behaviors and display empathy towards their peers. We know that in juvenile rats, absent or abnormal social play during juvenile development can result in deficiencies in adulthood, for example, social withdrawal and impaired social communication. The *Cacna1c* rat, therefore, is an ideal model to study the impact of gene alterations on the development of social behavior and communication, with importance to mood and neurodevelopmental disorders.

The drawback of this model is that a more regional and brain-site-specific deletion could provide better insight into the precise mechanisms and areas affected by the mutation causing the impairments. However, a site-specific deletion is not as translational as our current *Cacna1c* rat model.

What has surprised you the most while conducting your research?

Mutations in the *CACNA1C* gene have been linked to Timothy syndrome, which has characteristic features of autism spectrum disorder. Specifically, the affected children tend to have impaired

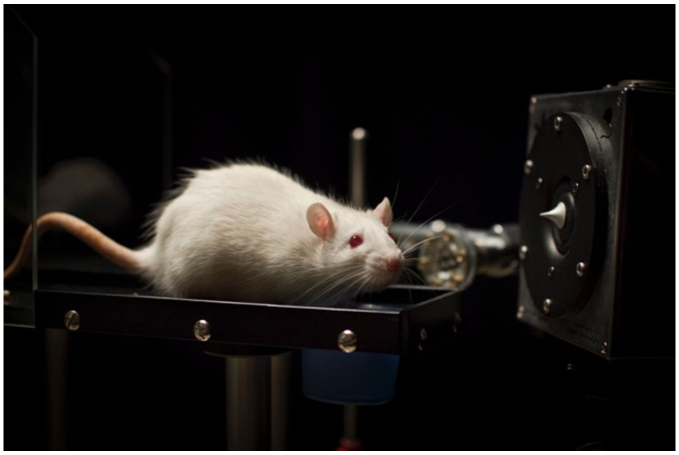

50-kHz ultrasonic vocalizations playback paradigm (Sprague Dawley rat).

communication and socialization skills, and delayed development of speech and language. Therefore, we were slightly surprised to find that while the rats exhibited deficits in the production of and response to ultrasonic communication, there were no obvious impairments in social play behavior or common autism-like phenotypes such as repetitive and stereotyped movements or self-grooming. Timothy syndrome rodent models and our *Cacna1c* rat model are two different mutations, each with specific effects on gene expression. In models of Timothy syndrome there is an increase in gene expression whereas in our model we have a decrease in gene expression. Therefore, this suggests that the specific expression levels for the *Cacna1c* gene may contribute differentially to the behavioral and communication deficits exhibited.

Describe what you think is the most significant challenge impacting your research at this time and how will this be addressed over the next 10 years?

Whenever studying social play and 50-kHz USV in rats it is always difficult to attribute any one specific vocalization to an individual rat, therefore we are not able to accurately say which rat is in fact vocalizing and when they are doing so. The ability to do this using minimally invasive, non-surgical techniques might provide more insight into the motivational properties behind the relationship of play and 50-kHz USV in rats. Additionally, during the analysis of our research, we noted that there is a lack of adequate software available to accurately analyze the acoustic characteristics and subtypes of rat 50-kHz USV. This means that the assessment needs to be done by a human observer, which is not a time- or cost-effective method. Machine learning software, however, continues to improve every year and some promising programs for mice already exist. It is my opinion, though, that this bottleneck in the analysis could be minimized within the next few years.

What changes do you think could improve the professional lives of early-career scientists?

I think that finding a way to provide an equal balance for women who wish to pursue a career in academics as well as have a family is very important. It seems that too often those who chose to have a family and a career in science are left to decide which is more important, leading many to leave academics altogether. Establishing better programs to support those with families, especially when they are international and have no family in the area, would be beneficial and could inspire more women to follow scientific careers and strive for the highly sought-after faculty positions. I also think that better overall guidance when you are nearing the end of your PhD would be advantageous to every new burgeoning postdoc. PhD students graduate and then are often times lost and aimless with no sense of direction for their future. I feel I have been very lucky in this regard and will get a postdoc position right away following my PhD defense. I can attribute this to good guidance and expertise from established faculty members and senior postdocs about what could be done next and what sort of steps are necessary in order to obtain grants or further experience. I think that this type of support structure would be a huge asset to all PhD students who wish to continue in scientific research careers.

What's next for you?

My interests are strongly tied to pro-social behavior and communication, and the deficiencies that result from interruptions during the normal developmental process. Based on our recent findings I will continue to explore deficits in social interactions as a result of specific gene alterations, and also gene and environmental manipulations. My aim is to discover approaches to rescue such impairments and help design translational treatment methods for the millions of people across the globe whose quality of life is severely impacted by deficits in social behavior and communication abilities.

Reference

Kisko, T. M., Braun, M. D., Michels, S., Witt, S. H., Rietschel, M., Culmsee, C., Schwarting, R. K. W. and Wöhr, M. (2018). *Cacna1c* haploinsufficiency leads to pro-social 50-kHz ultrasonic communication deficits in rats. *Dis. Model. Mech.* **11**: dmm034116, doi:10.1242/dmm.034116.
